# *MiR-148a* inhibits angiogenesis by targeting ERBB3^☆^

**DOI:** 10.1016/S1674-8301(11)60022-5

**Published:** 2011-05

**Authors:** Jing Yu, Qi Li, Qing Xu, Lingzhi Liu, Binghua Jiang

**Affiliations:** a Lab of Reproductive Medicine, Department of Pathology, Jiangsu Key Lab of Cancer Biomarkers, Prevention and Treatment, Cancer Center, Nanjing Medical University, Nanjing, Jiangsu 210029, China;; b Department of Pathology, Anatomy and Cell Biology, Thomas Jefferson University, Philadelphia, PA, USA.

**Keywords:** breast cancer, microRNA-148a, angiogenesis, ERBB3

## Abstract

MicroRNAs (miRNAs) play an important role in carcinogenesis in various solid cancers including breast cancer. Down-regulation of *microRNA*-*148a* (*miR*-*148a*) has been reported in certain cancer types. However, the biological role of *miR*-*148a* and its related targets in breast cancer are unknown yet. In this study, we showed that the level of *miR*-*148a* was lower in MCF7 cells than that in MCF10A cells. V-erb-b2 erythroblastic leukemia viral oncogene homolog 3 (*ERBB3*) is a direct target of *miR*-*148a* in human breast cancer cells through direct binding of *miR*-*148a* to *ERBB3* 3′-UTR region. Overexpression of *miR*-*148a* in MCF7 cells inhibited *ERBB3* expression, blocked the downstream pathway activation including activation of AKT, ERK1/2, and p70S6K1, and decreased HIF-1α expression. Furthermore, forced expression of *miR*-*148a* attenuated tumor angiogenesis *in vivo*. Our results identify *ERBB3* as a direct target of *miR*-*148a*, and provide direct evidence that *miR*-*148a* inhibits tumor angiogenesis through *ERBB3* and its downstream signaling molecules. This information would be helpful for targeting the *miR*-*148a*/*ERBB3* pathway for breast cancer prevention and treatment in the future.

## INTRODUCTION

Breast cancer is the most common cancer in women with more than 400,000 deaths each year worldwide. MicroRNAs (miRNAs) are a class of small noncoding RNAs that have been identified as a new kind of gene expression regulators through targeting the 3′-untranslated region (UTR) of mRNAs for translational repression or degradation[1],[2]. MiRNAs are differentially expressed in various human cancers, functioning either as oncogenes or tumor suppressors by controlling the expression of their target genes[3],[4]. Some miRNAs were related to cancer biopathological features like vascular invasion, lymph node metastasis, tumor stage and cell proliferation[3]–[5]. Recent studies have shown that reduced expression of *miR*-*148a* occurs in chronic lymphocytic leukemia, gastrointestinal cancer, and esophageal carcinoma[5]–[8] and is correlated with the outcome of certain malignancies[8],[9]. The hypermethylation-associated *miR*-*148a* silencing has also been reported to be correlated with human cancer metastasis[10]. Overexpression of *miR*-*148a* attenuated paclitaxel resistance of hormone-refractory, drug-resistant prostate cancer PC3 cells by regulating mitogen- and stress-activated kinase 1 (MSK1) expression[11]. Known direct targets of *miR*-*148a* include transcription growth factor-β-induced factor 2 (TGIF2), DNA (cytosine-5-)-methyltransferase 3β (DNMT3b), pregnane X receptor (PXR), neddylation-dissociated 1, and DNA methyltransferase-1[10],[12]–[15]. However, the biological roles of *miR-148a* and the target genes in breast cancer have not yet been defined.

V-erb-b2 erythroblastic leukemia viral oncogene homolog 3 (ERBB3) is a member of the epidermal growth factor receptor (EGFR) family, which consists of EGFR/ERBB1, ERBB2/Neu/HER2, ERBB3, and ERBB4. Since ERBB3 lacks intrinsic kinase activity, signal transduction occurs through formation of heterodimers with ERBB2 and other members. The binding of peptides of the EGF-related growth factor family to the extracellular domain of the ERBB receptors causes the formation of homo- and heterodimers. Ligand binding induces intrinsic receptor kinase activity and then stimulates intracellular signaling cascades, including the phosphatidylinositol-3 kinase (PI3K)/AKT and MAPKs cascade[16],[17]. Elevated expression of ERBB3 is frequently observed in breast cancer, which may play an important role in breast cancer progression and chemotherapy resistance[18],[19]. Recent studies have demonstrated that *miR*-*125* and *miR*-*205* act as tumor suppressors through directly targeting *ERBB3*[20],[21].

Tumor angiogenesis is required for tumor development and growth in which hypoxia-inducible factor 1α (HIF-1α) plays a pivotal role[22],[23]. In the present study, we want to identify novel target(s) of *miR*-*148a*, which may be related to tumor angiogenesis, identify signaling pathways that are regulated by *miR*-*148a*, and determine the direct role of *miR*-*148a* in angiogenesis. This will provide direct evidence for the role and potential mechanism of *miR*-*148a* in regulating breast tumor angiogenesis.

## MATERIALS AND METHODS

### Cell culture and reagents

Human breast cancer cells MCF7 and embryonic kidney cells HEK293 were cultured in Dulbecco's Modified Eagle Media (DMEM) supplemented with 10% fetal bovine serum, 100 units/mL penicillin, 100 mg/mL streptomycin, and 5% CO_2_ at 37°C. ERBB3 antibodies were purchased from Bioworld Technology (Louis Park, MN, USA). Antibodies against phospho-AKT, AKT, phospho-ERK1/2, ERK, phospho-p70S6K1 and p70S6K1 were purchased from Cell Signaling Technology (MA, USA), Antibodies against HIF-1α was from BD Biosciences (NJ, USA). Antibodies against GAPDH were from Kang-Cheng (Shanghai, China). Puromycin and β-actin monoclonal antibody were from Sigma (St. Louis, MO, USA). Matrigel was from BD Biosciences (Franklin Lakes, NJ, USA). Trizol was purchased from Invitrogen (Carlsbad, CA, USA).

### Lentivirus packaging and stable cell lines establishment

The lentiviral vectors with RFP tag carrying scrambled miRNA (pLe-miR-SCR), *miR*-*148a* (pLe-miR-148a), and packaging kit were purchased from Thermo Scientific (Huntsville, AL, USA). Lentiviral packaging was performed using HEK293T cells by following the manufacturer's manual. MCF7 cells were infected with lentiviruses, and selected by puromycin to obtain stable cell lines expressing *miR*-*SCR* and *miR*-*148a*.

### RT-PCR and *Taq*Man real-time PCR analysis of *miR*-*148a*

Total RNAs of MCF7 cells were extracted using Trizol reagent according to the manufacturer's instruction. The stem-loop RT-PCR assay was used to quantify the miRNA expression levels as was described previously[35],[36]. The PCR primers used were as follows: *miR*-*148a* RT primer: 5′-CTCAACTGGTGTCGTGGAGTCGGCAATTCAGTTGAGACAAAGTT-3′. *miR*-*148a* PCR primers: sense: 5′-ACACTCCAGCTGGGTCAGTGCACTACAGAA-3′; anti-sense: 5′-TGGTGTCGTGGAGTCG-3′. *U6* RT primer: 5′-TGGTGTCGTGGAGTCG-3′. *U6* PCR primers: sense: 5′-CTCGCTTCGGCAGCACA-3′; anti-sense: 5′-AACGCTTCACGAATTTGCGT-3′. PCR products were separated on 1.5% agarose gels, stained with ethidium bromide, and visualized under UV light. The levels of *miR*-*148a* in MCF7, T47D and MCF10A cells were tested using *Taq*Man real-time PCR Kit (Applied Biosystems, Carlsbad, CA, USA) according to the instruction. The levels of *U6* were used as an internal control.

### Transient transfection and luciferase assay

Reporter plasmids containing *ERBB3* 3′ UTR region with *miR*-*148a* binding site (wild type) or mutation binding site (mutant) were cloned into pMIR-REPORTER. HEK293 cells were seeded in 24-well plates and cultured overnight. To investigate whether miR-148a directly regulates *ERBB3* transcriptional expression by binding to the *miR*-*148a* binding site of *ERBB3*, pre-miR-148a or pre-miR-SCR was co-transfected with plasmid pGL4.74 expressing renilla luciferase and the *ERBB3* wild type or mutant reporter plasmid. After cultured for 48 h, cells were lysed with passive lysis buffer (Promega, WI, USA), renilla and firefly luciferase activities were measured using the dual luciferase assay system (Promega WI, USA). *ERBB3* transcriptional activity was normalized to renilla luciferase activity, which was an internal control for transfection efficiency. Each experiment was repeated at least three times.

### Immunoblotting assay

Cells were harvested and lysed on ice for 30 min in RIPA buffer (150 mmol/L NaCl, 100 mmol/L Tris, pH 8.0, 0.1% SDS, 1% Triton X-100, 1% sodium deoxycholate, 5 mmol/L EDTA and 10 mmol/L NaF) supplemented with 1 mmol/L sodium vanadate, 2 mmol/L leupeptin, 2 mmol/L aprotinin, 1 mmol/L phenylmethylsulfonyl fluoride (PMSF), 1 mmol/L DTT, and 2 mmol/L pepstatin A. The lysates were centrifugated, and the supernatants were collected as total cellular protein extracts. The total protein extracts were separated by SDS-polyacrylamide gel electrophoresis (SDS-PAGE), and transferred to nitrocellulose membranes in transfer buffer (20 mmol/L Tris-HCl, pH 8.0; 150 mmol/L glycine; 20% (v/v) methanol). Membranes were blocked with 5% nonfat dry milk in 1×PBS containing 0.05% Tween 20, and incubated with specific antibodies. The protein bands were detected by incubation with horseradish peroxidase-conjugated antibodies, and visualized with the SuperSignal West Pico Chemiluminescent Substrate Kits (Thermo Scientific, MA, USA).

### Angiogenesis assay on chicken chorioallantoic membrane (CAM)

Fertilized chicken eggs were purchased from SPAFAS (Preston, CT, USA), and incubated at 37°C with 70% humidity. On day 8, an artificial air sac was created and a small window in the shell over the artificial air sac was cut as we described[37]. MCF7-miR-148a or MCF7-miR-SCR stable cells were resuspended in serum-free medium, and mixed with equal volume of Matrigel. Then, aliquots (3×10^6^, 40 µL) of the mixture were applied onto the CAM of 9-d-old embryos. The area around the implanted Matrigel was photographed 5 d after the implantation, and the number of blood vessels was obtained by counting the branching of blood vessels. Assays for each treatment were carried out using 8 chicken embryos.

### Cell proliferation assay

To investigate the effects of *miR*-*148a* on the cell growth of MCF7 cells, the stable cell MCF7-miR-SCR and MCF7-miR-148a were seeded in a 96-well plate (1,000 cells per well) and incubated at 37°C in 5% CO_2_ incubator. The proliferation of the cells was measured using a proliferation assay kit, CCK8 kit (Dojindo Laboratories, Kumamoto, Japan) according to the manufacturer's instruction. Results were obtained from three independent experiments.

### Statistical analysis

All values in the present study were reported as mean ±SE. Student's unpaired *t* test was used for statistical analyses. Differences between values were considered significant at *P* < 0.05.

## RESULTS

### *MiR*-*148a* expression in breast cancer cells

**Fig. 1:**
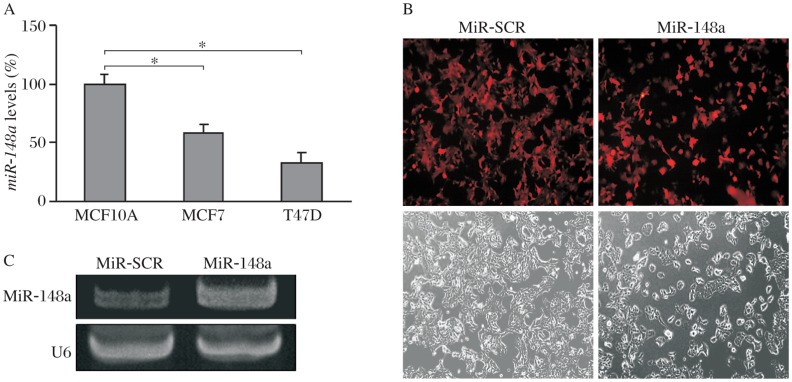
Endogenous and forced expression of *miR-148a* in breast cancer cells. A: Total RNAs from human breast cancer cells MCF7 and T47D and immortalized normal breast epithelial cells MCF10A were subjected to *Taq*Man real-time RT-PCR analysis for *miR*-*148a* expression, *P < 0.05. B: MCF7 cells were infected by lentivirus carrying scrambled miRNA precursor (SCR) or *miR*-*148a*, and cultured in the medium containing puromycin to select and obtain stable cell lines. The red images on the left panels showed the expression of the lentivirus in the cells. C: *MiR*-*148a* expression levels in the cells were analyzed by stem-loop RT-PCR assay, and *U6* expression levels were used as an internal control for RNA loading.

To determine whether the expression of *miR*-*148a* is downregulated in breast cancer cells, human breast cancer cell lines MCF7 and T47D and the immortalized normal breast epithelial cells MCF10A were used to test the levels of *miR*-*148a* by *Taq*Man real-time RT-PCR analysis. The results showed that *miR*-*148a* expression was significantly decreased in MCF7 and T47D cells (*P* < 0.05, ***Fig. 1A***). To investigate the role of *miR*-*148a* in breast cancer, MCF7 cells were infected with lentiviruses expressing *miR*-*148a* and *miR*-*SCR* control, and selected by puromycin to obtain stable cell lines. We established stable cells with fluorescent and contrast phase representative pictures of MCF7-miR-SCR and MCF7-miR-148a cells (***Fig. 1B***). Compared to MCF7-miR-SCR, the *miR*-*148a* level in MCF7-miR-148a was much higher (***Fig.1C***). These results indicated that we successfully established the stable breast cancer cell lines expressing *miR*-*SCR* and *miR*-*148a*, respectively.

### *ERBB3* acting as a direct target of miR-148a

**Fig. 2:**
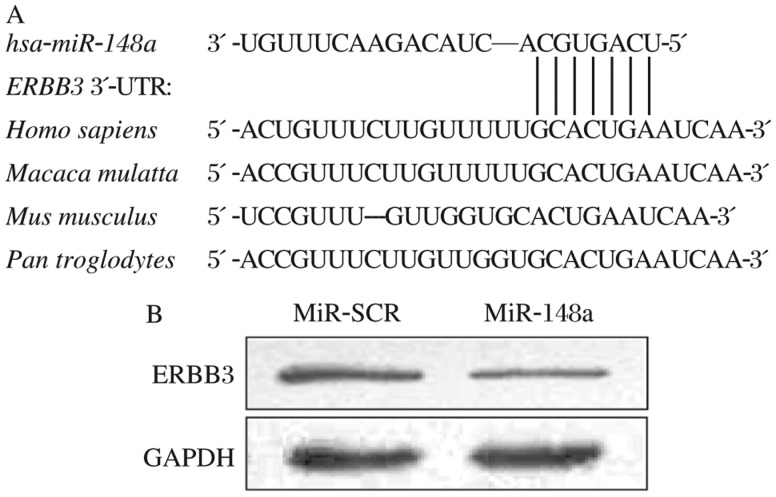
*MiR*-*148a* targeted to *ERBB3*. A: Putative binding site of *ERBB3* 3′-UTR by *miR*-*148a*. The seed-matching sites were predicted by PicTar, TargetScan and FindTar software, and were marked in red. DNA sequences of potential *miR*-*148a* binding site predicted within the human *ERBB3* 3′-UTR were also highly conserved among other species. B: MCF7 cells expressing scramble miRNA precursor (SCR) or *miR*-*148a* were used for Western blot analysis to determine the levels of ERBB3 expression with GAPDH as an internal control.

**Fig. 3:**
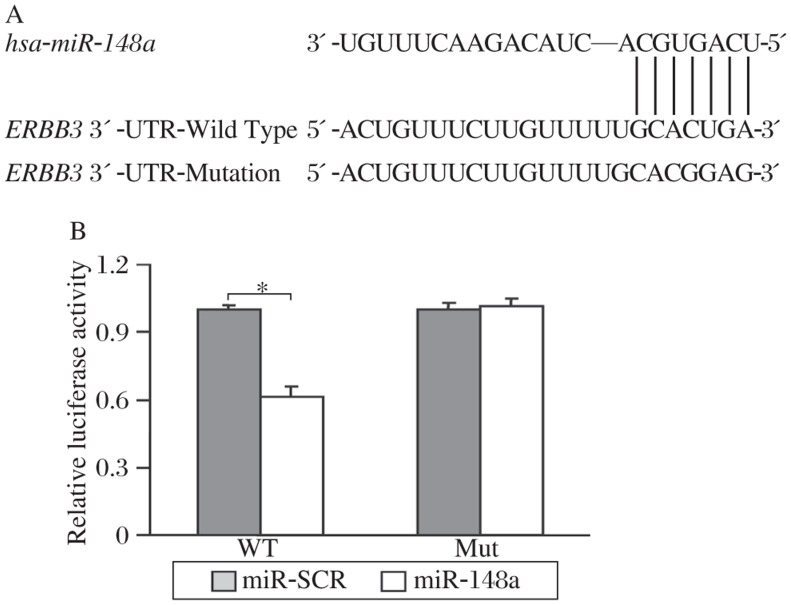
*MiR-148a* interacted with *ERBB3* 3′-UTR to inhibit its transcriptional activation. A: Schematic illustration of *ERBB3* 3′-UTR wild type (WT) and mutant reporter constructs with the seed-matching sites marked in red. B: MCF7 cells were transfected with *ERBB3* 3′-UTR wild type (WT) and mutant reporter constructs in the presence of scramble miRNA precursor (SCR) or *miR*-*148a*, and the cells were cultured for 36 h after the transfection. Relative luciferase activities were measured and calculated as the ratio of firefly/renilla activities in the cells, and normalized to those of the SCR control. The results were presented as means±SE from three independent experiments, *P < 0.05.

To determine the direct target gene(s) of *miR*-*148a*, we identified the potential binding sites of *miR*-*148a* using the combination of PicTar, TargetScan and FindTar software, and found that *ERBB3* was one of the predicted targets that contain putative conserved *miR*-*148a* binding sites within its 3′UTR. The binding site of *miR*-*148a* in *ERBB3* 3′-UTR region is highly conserved among several different species (***Fig. 2A***). To test whether *miR*-*148a* regulates *ERBB3* expression at protein level, total proteins prepared from MCF7-miR-148a cells and MCF7-miR-SCR cells were analyzed by Western blotting. Overexpression of *miR*-*148a* greatly decreased ERBB3 expression in MCF7-miR-148a cells (***Fig. 2B***), suggesting that ERBB3 is a downstream target of *miR*-*148a*. To further determine whether *ERBB3* is a direct target of *miR*-*148a*, we constructed luciferase reporters containing *ERBB3* 3′-UTR regions and the predicted binding site of *miR*-*148a* (WT, ***Fig. 3A***), or the predicted binding site with the mutation in the seed sequence (Mut, ***Fig. 3A***). MCF7 cells were transfected with the *ERBB3* reporter plasmids in the absence or presence of lentivirus plasmids carrying *miR*-*148a* and *miR*-*SCR*. We found that *miR*-*148a* significantly inhibited *ERBB3* wild type reporter activities, but not the mutant reporter activity (***Fig. 3B***). This result shows that *miR*-*148a* inhibits *ERBB3* wild type reporter activities through the binding of *miR*-*148a* in the seed region. These results demonstrate that *ERBB3* is a direct target of *miR*-*148a* in breast cancer cells.

### *MiR-148a* inhibited the activation of AKT, ERK, and p70S6K1 and decreased HIF-1α expression.

**Fig. 4:**
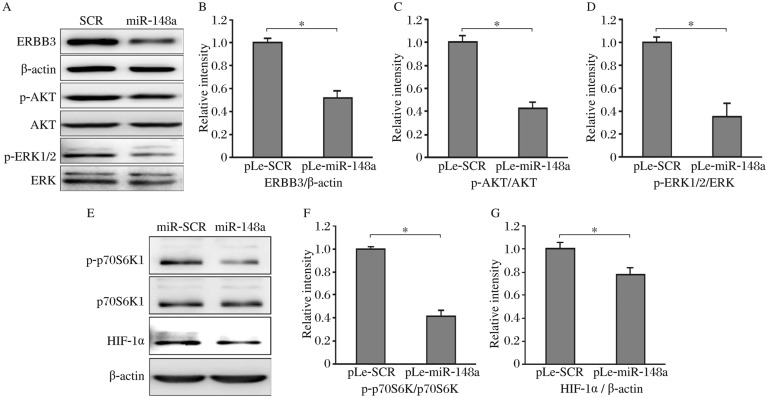
*MiR-148a* inhibited AKT and ERK activation, and HIF-1α expression. Stable breast cancer cells expressing scramble miRNA precursor (SCR) or *miR*-*148a* were obtained by puromycin selection. A:The protein levels of ERBB3, β-actin, p-AKT, total AKT, p-ERK1/2, and total ERK were analyzed by Western blotting. The relative densities of total ERBB3 (B), p-AKT (C), and p-ERK1/2 (D) levels were quantified and analyzed by Image J software from three replicate experiments, *P < 0.05. E: The protein levels of p-p70S6K1, total p70S6K1, HIF-1α, and β-actin were analyzed by Western blotting. The relative densities of p-p70S6K1 (F) and HIF-1α (G) levels were quantified and analyzed by Image J software from three independent experiments, *P < 0.05.

AKT and ERK signaling pathways are two key downstream pathways of ERBB3, and play important roles in angiogenesis and cancer development[24],[25]. To investigate the effect of *miR*-*148a* on their activation, we showed that levels of phospho-AKT (p-AKT) and phospho-ERK1/2 (p-ERK1/2) were greatly suppressed in the cells expressing *miR*-*148a* (***Figs. 4A to 4D***), indicating that *miR*-*148a* inhibited ERBB3 expression with functional role by decreasing downstream AKT and ERK activation. Furthermore, *miR*-*148a* overexpression inhibited p70S6K1 activation and HIF-1α expression (***Fig. 4E to 4G***). Given the pivotal roles of these ERBB3 downstream molecules in regulating tumor growth and angiogenesis, these results indicate that *miR*-*148* may inhibit tumor angiogenesis *via* targeting ERBB3 and its downstream molecules.

### Overexpression of *miR*-*148a* inhibited tumor angiogenesis *in vivo*

**Fig. 5:**
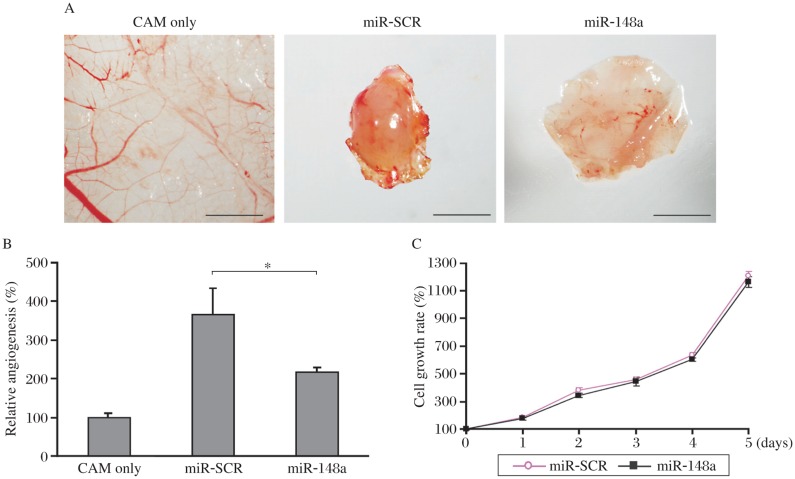
*MiR*-*148a* inhibited tumor angiogenesis *in vivo*. A: MCF7 cells expressing lentivirus with scrambled miRNA precursor (*miR*-*SCR*) or *miR*-*148a* were used for angiogenesis assay in the CAM. The angiogenesis responses were analyzed 5 days after the implantation of the MCF7 cells. The representative photos of the CAM, the cells expressing *miR*-*SCR* or *miR*-*148a*. Bar, 2 mm. B: Relative angiogenesis responses in the CAM were calculated and analyzed from eight independent CAM tissues, and presented as means±SE (*P < 0.05). C: Cell proliferation assay of MCF7-miR-SCR and MCF7-miR-148a cells was performed using CCK8 kit.

In order to study whether overexpression of *miR*-*148a* inhibits tumor angiogenesis, MCF7 cells expressing *miR*-*148* and *miR*-*SCR* were resuspended in serum-free medium and used for angiogenesis assay on the chicken CAM of 9-d-old chicken embryos. The angiogenesis responses were analyzed on the CAM 4 d after the implantation. MCF7 breast cancer cells induced the angiogenesis responses 4-fold higher than the normal angiogenesis in the CAM, and the forced expression of *miR*-*148a* significantly inhibited cancer cell-inducing angiogenesis responses by 45% (***Fig. 5B***). Meanwhile, cell proliferation assay indicated that the growth rate of MCF7-miR-SCR was similar to that of MCF7-miR-148a, indicating that the angiogenesis response was not due to the effect of *miR*-*148a* overexpression on cell proliferation (***Fig. 5C***). These results suggest that overexpression of *miR*-*148a* specifically inhibits angiogenesis.

## DISCUSSION

MiRNAs act as tumor suppressors or oncogenes in a variety of cancers including breast cancer. Angiogenesis is the process by which new blood capillaries are generated from the pre-existing vasculature. Angiogenesis is important for tumor growth, development, and metastasis since tumors cannot grow larger than 1-2 mm in diameter without an efficient blood supply[26]. Growing evidence demonstrates that miRNAs such as *miR*-*378*, *miR*-*296*, and the *miR*-*17∼92* cluster can regulate angiogenesis[27]–[29]. However, the role of *miR*-*148a* in tumor angiogenesis is unknown. ERBB3 is involved in regulating cancer initiation, tumor metastasis and angiogenesis and is important in breast cancer development[30],[31]. In addition, ERBB3 is involved in acquired resistance to chemotherapy[32],[33]. In this study, we demonstrated that the level of *miR*-*148a* in breast cancer cells MCF7 was lower than that in immortalized normal breast epithelial cells MCF10A, and *ERBB3* is a direct target of *miR*-*148a*. This data show that *miR*-*148a* is a new regulator of ERBB3 in cancer cells, and the downregulation of *miR*-*148a* is a novel molecular mechanism for breast cancer development.

ERBB3 can heterodimerize with ERBB2 to activate AKT and ERK signaling pathways in different cells. Consistent with ERBB3 inhibition, forced expression of *miR*-*148a* in breast cancer cells inhibited the activation of ERBB3 downstream molecules including AKT, ERK1/2, and p70S6K1. *MiR*-*148a* also suppressed HIF-1α expression, which is a rate-limiting subunit for forming functional HIF-1 transcription factor. HIF-1 regulates the expression of VEGF and other angiogenesis regulators[34]. Thus, we hypothesize that *miR*-*148a* inhibits breast tumor angiogenesis. To support this hypothesis, we demonstrated that *miR*-*148a* overexpression attenuated breast tumor angiogenesis induced by MCF7 cells. In summary, this study demonstrates that *miR*-*148* directly targets *ERBB3* through the seed sequence binding site at its 3′-UTR. Reduced expression of *miR*-*148a* in breast cancer cells leads to the elevation of ERBB3 expression to activate AKT and ERK1/2 signaling pathways, which may in turn increase p70S6K1 activation and HIF-1α expression. *miR*-*148a* attenuates angiogenesis likely through directly inhibiting ERBB3 for transmitting the signals to its downstream signaling molecules.Our findings suggest that *miR*-*148a*/*ERBB3* pathway would be a promising therapeutic target for breast cancer in the future.
